# Study on the Preparation and Properties of MT-GE (6S-5-Methyltetrahydrofolate Calcium Salt Crystal Form C-Gelatin) Nanofiber Membrane

**DOI:** 10.3390/gels12070563

**Published:** 2026-06-25

**Authors:** Yuhang Wang, Ke Wang, Mochi Zhu, Yu Liu, Tianyue Xu, Rui Duan, Junjie Zhang

**Affiliations:** 1 School of Ocean Food and Bioengineering, Jiangsu Ocean University, Lianyungang 222005, China; 2 Jiangsu Institute of Marine Resources Development, Jiangsu Ocean University, Lianyungang 222005, China; 3 School of Marine Science and Fisheries, Jiangsu Ocean University, Lianyungang 222005, China

**Keywords:** 6S-5-methyltetrahydrofolate calcium salt crystal form C, gelatin, nanofiber membrane

## Abstract

Folate is an essential vitamin associated with protein and DNA synthesis in the body. Compared with synthetic folic acid, 6S-5-methyltetrahydrofolate calcium salt crystal form C (MTHF CAC) is safer and has a higher bioavailability. In this study, a nanofiber membrane (MT-GE) was prepared from fish gelatin and MTHF CAC in the aqueous system via electrospinning. Differential scanning calorimetry showed higher transition temperatures for MT-GE than for GE. The weight loss curve of MT-GE detected by thermogravimetric analysis was higher than that of GE. The results corresponded to those of X-ray diffraction, which indicated the slightly higher crystalline strength of MT-GE than GE. Therefore, the inclusion of MTHF CAC improved the physical characteristics of GE nanofibers. High-performance liquid chromatography analysis revealed that the retention of MTHF CAC in MT-GE reached 85.57%, which suggested that electrospinning caused no effect on the properties of MTHF CAC. The MT-GE membrane supported cell proliferation, and the Cell Counting Kit-8 results indicated that the cell proliferation rate exceeded 100%, with the MT-GE solution demonstrating more than double the proliferation rate of the control group. Therefore, MT-GE has great potential for use as a medical biomaterial.

## 1. Introduction

Electrospinning is a technique where polymer solutions or molten polymers are stretched into nanofibers by a high-voltage electrostatic field [1,2]. Nanofiber membranes have a huge specific surface area and porosity [3,4,5], as well as a high degree of designability in composition and structure [6]. Compared with other film-forming techniques such as solution casting, phase separation, and 3D printing, electrospinning can directly prepare nanofiber networks of biomimetic extracellular matrices and has unique advantages in creating membrane structures with high porosity and high specific surface area, especially suitable as a preparation platform for tissue engineering scaffolds and active substance carriers [7]. In this study, MTHF CAC, a new type of folate and an essential bioactive substance, was loaded into the electrospun membrane, which may provide valuable information for tissue engineering applications.

Gelatin is approved by the Food and Drug Administration as one of the most commonly used biopolymers due to its excellent biocompatibility, degradability, and wide applicability [8]. At present, the gelatin used in electrospinning mainly originates from the skins of porcine and cattle. Nanofiber membranes, which are produced by electrospinning a gelatin solution derived from porcine skin and 2,2,2-trifluoroethanol as the solvent, exhibit exceptional thermal stability and mechanical properties, which make them a promising scaffold material for tissue engineering [9]. The nanofiber mats made using cattle skin gelatin show a positive effect on the cell proliferation of human dermal fibroblast and embryonic kidney cells [10].

Compared with gelatin of mammalian origin, fish gelatin has a lower zoonotic risk and fewer religious restrictions [11,12,13,14], and recent studies confirm that it performs better in terms of cell adhesion and biocompatibility [15]. An et al. fabricated nanofibers from channel catfish skin gelatin via electrospinning technology successfully [11]. Arfat et al. developed ZnO nanocomposite antibacterial films based on a blend system of fish protein isolate (FPI) and tilapia fish skin gelatin (FSG) [12]. Padrão et al. systematically investigated the effects of glutaraldehyde vapor crosslinking on the morphology, swelling behavior, barrier properties, and cytocompatibility of two types of fish gelatin electrospun membranes, confirming their application potential as wound dressings and tissue engineering scaffolds [13], and further developed an all-protein-based, metal-free nanoparticle electrospun antibacterial membrane based on a composite system of fish gelatin and bovine lactoferrin (bLF) [14]. Kwak produced ultrafine nanofiber webs using an aqueous solution of cold-water fish gelatin through electrospinning [16]. The nanofibers demonstrated higher cell adhesion and proliferation rates compared with those made from mammalian gelatin. Songchotikunpan developed an acidic system of ultrafine fish-gelatin nanofibers that were subsequently treated with glutaraldehyde [17]. The resulting nanofibrous membranes exhibited improved water resistance and showed a high potential for use in the biomedical industry.

Folate is an essential vitamin associated with protein and DNA synthesis in the body and serves as a methyl donor in one-carbon metabolism [18,19]. Currently, in addition to synthetic folic acid, crystalline forms of folate include 6S-5-methyltetrahydrofolate calcium salt crystal form C (MTHF CAC), (6S)-5-methyltetrahydrofolic acid, and glucosamine salt. Compared with synthetic folic acid, MTHF CAC is safer and more available to the body [20,21]. Zhou reported that folic acid protected against delayed healing of skin wounds in mice with diabetes and contributed to tissue regeneration [22,23]. Bagheri pointed out that the main reason for slow wound healing in diabetic patients was the lack of NO in the body. NO was a critical signaling molecule essential for normal wound repair. Folate had the homocysteine-lowering effects and antioxidant actions, which had a healing effect on diabetic wounds [24]. Hybrid electrospinning of porcine skin gelatin and synthetic folic acid was used to produce a fibrous membrane with excellent biocompatibility for tissue engineering and wound healing [25]. However, the reports on the electrospun membrane prepared from MTHF CAC and gelatin were very limited. Until now, no studies have been found on the fabrication of electrospun membranes using MTHF CAC and fish gelatin. Most reported studies used synthetic folic acid, which needs to undergo a metabolic process in the liver, and organic solvents to prepare an electrospinning solution, which limits its potential for clinical application [24,25].

The traditional electrospinning is usually employed with organic solvents [25,26,27], but reducing the harm of organic reagents is a trend in the production of biomaterials [28]. Zulkifli explicitly listed the six most common toxic solvents used in electrospinning and their cytotoxicity grades, among which hexafluoroisopropanol and trifluoroethanol (TFE) exhibited the strongest cytotoxicity toward fibroblasts. Residual solvents damaged cell membrane integrity, interfered with cellular metabolism, and ultimately reduced cell proliferation rates by more than 50% [29]. Conventional toxic organic solvents used in electrospinning (such as HFIP, TFE, N,N-dimethylformamide (DMF) and dichloromethane (DCM)) produced volatile organic compounds (VOCs) during fabrication. Notably, the high specific surface area and dense structure of nanofibers hindered complete solvent evaporation. Even trace amounts of residual solvents exerted severe cytotoxicity toward cells and tissues, significantly inhibiting cell proliferation and inducing cell apoptosis [7].

Since MTHF CAC was insoluble in the organic solvents, in the present study, the nanofiber membrane (MT-GE) with the components of fish gelatin and MTHF CAC was prepared in the aqueous system through electrospinning. Its morphological characteristics, thermal properties, and crystal structure were investigated, and its cytotoxicity was evaluated. This research provides basic information on the characteristics of MT-GE which could be a new kind of biomaterial for medical applications.

## 2. Results and Discussion

### 2.1. Morphological Characteristics

**Figure 1:**
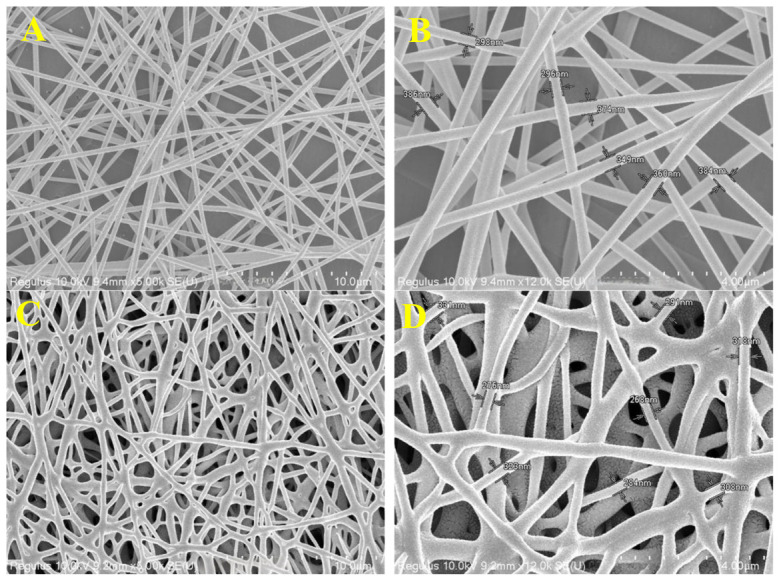

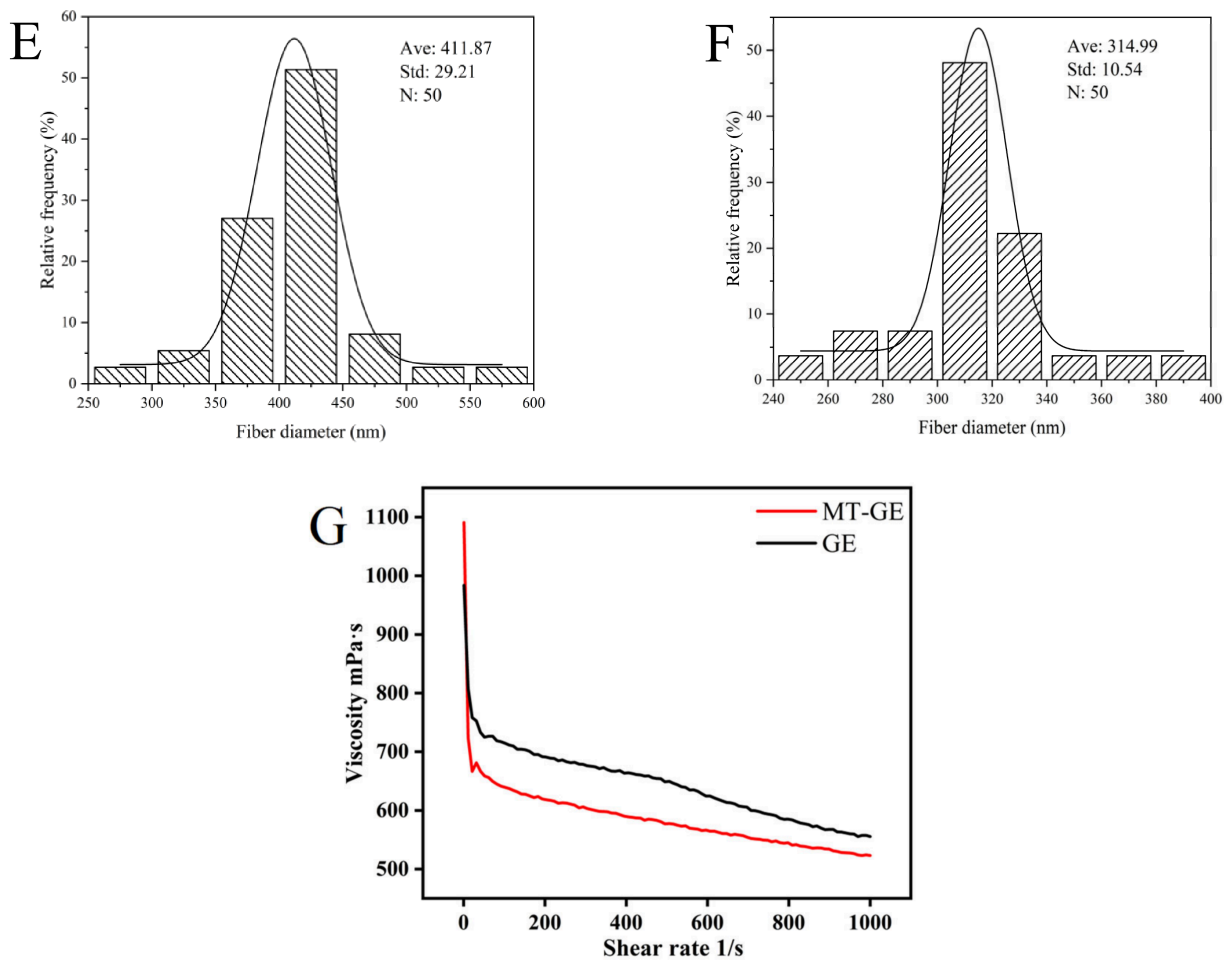
SEM results and diameter distribution statistics of MT-GE and GE. (**A**) GE nanofibers (magnification: 5 KX, scale bar: 10 µm). (**B**) GE nanofibers (magnification: 12 KX, scale bar: 4 µm). (**C**) MT-GE nanofibers (magnification: 5 KX, scale bar: 10 µm). (**D**) MT-GE nanofibers (magnification: 12 KX, scale bar: 4 µm). (**E**) Statistical results of GE diameter distribution. (**F**) Statistical results of MT-GE diameter distribution. (**G**) Shear rate-viscosity curve.

Figure 1 shows the scanning electron microscopy (SEM) and diameter distribution results of MT-GE and GE. The GE nanofibers had a beadless and smooth surface (Figure 1A) while MT-GE had a rough mesh structure that was formed (Figure 1C). The SEM results showed that the diameters of GE fiber and MT-GE were mainly distributed in the ranges of 411.87 ± 29.21 nm and 314.99 ± 10.54 nm, respectively (Figure 1E,F). The difference in the morphology and diameters of the two samples was mainly attributed to the dissimilarity of the rheological properties of the electrospun solution (Figure 1G). The gelatin solution was a non-Newtonian fluid and its viscosity was determined by the shear rate. The viscosity of the solution decreased with increasing shear rate, which exhibited shear-thinning behavior [30,31,32]. The MT-GE blends were 5.67–12.09% less viscous than GE for shear rates in the range of 200–1000 s^−1^.

**Figure 2:**
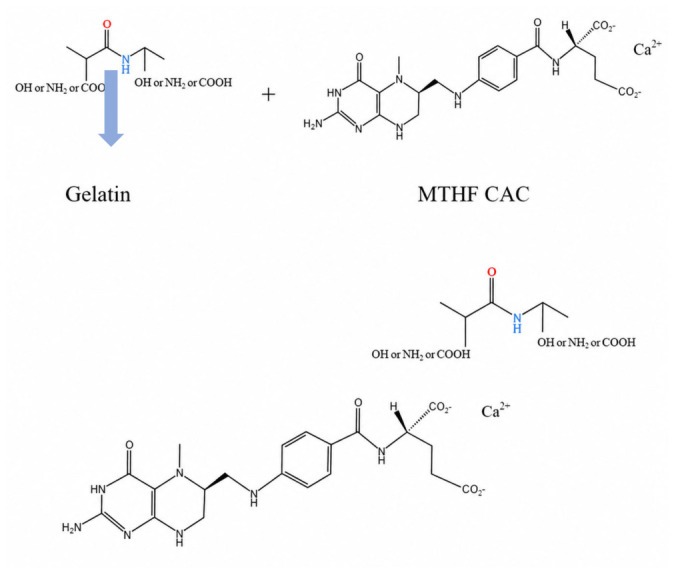
Mechanism reaction of gelatin with MTHF CAC. Red and blue highlight oxygen and nitrogen atoms of peptide bonds in gelatin, respectively.

In the e-spinning process, as the viscosity of the spinning solution decreased, the evaporation rate would increase. The fibers could be cured and shaped faster in air, resulting in the formation of finer fibers [32]. However, if the solvent evaporated too fast, insufficient stretching would occur and the curved and irregular fibers would form [33]. The decrease in MT-GE viscosity might be due to the fact that the calcium ions in the MTHF CAC solution could bridge the negatively charged carboxyl groups in the gelatin (Figure 2), which reduced the electronic repulsion [34,35]. Therefore, compared with the spinning solution of GE, that of MT-GE evaporated faster; thus, the electrospun fibers had a smaller diameter and lower uniformity.

### 2.2. Fourier Transform Infrared Spectroscopy (FTIR)

The FTIR spectra of MT-GE and GE are shown in Figure S1. MT-GE and GE had major characteristic peaks in the amide A band at 3285 cm^−1^, in the amide I band at 1647 cm^−1^, in the amide II band at 1539 cm^−1^, and in the amide III band at 1241 cm^−1^ and in the amide IV band at 653 cm^−1^. The amide A and I-III bands were the major characteristic peaks that prove the peptide chain backbone structures of a protein [36]. The formation of hydrogen bonds caused the wave number of the amide A band (3400–3440 cm^−1^) to blueshift to about 3300 cm^−1^ [37,38]. The slight decrease in transmittance of the band at 3285 cm^−1^ corresponding to the -OH functional group might indicate the formation of hydrogen bonds between gelatin and MTHF CAC [39,40]. The strength of the amide bonds of MT-GE was weaker than that of GE (reduced peak heights in the amide A, amide I, amide II, amide III and amide IV bands), which might be because the -COOH groups in the gelatin molecule form ionic bonds with calcium ions in the MTHF CAC (Figure 2), providing nucleation sites for inorganic crystal growth.

The FTIR spectra of PLLA/catfish skin gelatin nanofibers showed that with the increase in PLLA proportion in the electrospinning solution, the molecular interactions between gelatin and PLLA became weaker [11]. This might be attributed to the fact that PLLA lacked -OH groups binding to -NH2 groups in gelatin to form hydrogen bonds. In contrast, fish gelatin contained a large amount of -OH, -NH_2_, and -COOH, and MTHF CAC molecules contained -COOH and -OH groups. Gelatin and MTHF CAC could form a lot of hydrogen bonds, which led to the further formation of a relatively stable structure of MT-GE.

### 2.3. Differential Scanning Calorimetry and Thermogravimetry Analysis (DSC and TGA)

**Figure 3:**
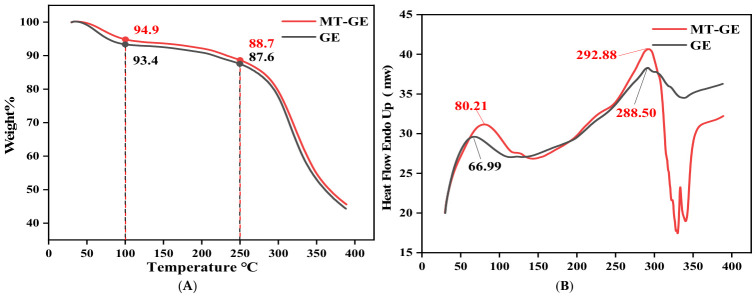
TGA and DSC curves of MT-GE and GE. (**A**) TGA curves of MT-GE and GE. vertical dotted lines mark the 100 °C and 250 °C boundary temperatures of the three thermal decomposition stages. (**B**) DSC curves of MT-GE and GE.

Figure 3A showed the TGA results for MT-GE and GE. The thermal decomposition of GE and MT-GE occurs in three stages: the first stage is around 50–100 °C, the second stage is around 100–250 °C, and the third stage is above 250 °C and exhibits continuous mass loss.

In stage 1 (50–100 °C), the sample experienced rapid mass loss, mainly due to the evaporation of free water and weakly bound water physically adsorbed in the nanofiber membrane, along with the initial depolymerization of the gelatin macromolecular network. At 100 °C, the residual masses of MT-GE and GE were 94.9% and 93.4% respectively. The mass loss of MT-GE was lower, indicating the additional hydrogen bonds formed between MTHF CAC and gelatin molecules which bind more water molecules firmly within the composite network structure, making evaporation more difficult. During Phase 2 (100–250 °C), the rate of mass loss of the sample significantly slows down. This process corresponds to the detachment of the strongly bound crystalline water in the gelatin molecules, the gradual destruction of the secondary structure of the peptide chains, and the breakage of weak hydrogen bonds between molecules. At 250 °C, the residual masses of MT-GE and GE were 88.7% and 87.6% respectively. MT-GE still maintained a higher residual mass, further confirming the enhanced intermolecular interaction between MTHF CAC and gelatin. Phase 3 was above 250 °C, and the samples underwent continuous and rapid mass loss. This was mainly due to the breakage of the covalent bonds in the main chain of the gelatin peptide chains, the thermal decomposition of amino acid residues, and the complete degradation of the macromolecular structure into small volatile products. At approximately 340 °C, the thermal decomposition rate of the samples slowed down significantly with a reduced slope of the mass loss curves, indicating the completion of the main thermal decomposition reactions. Correspondingly, the weight loss curve of MT-GE was slightly higher than that of GE at all tested temperatures, indicating that the thermal stability of MT-GE was marginally superior to that of GE. However, since the loading amount of MTHF CAC was only 0.4% (*w*/*w*), the compositional difference between the two samples was little, and thus their thermogravimetric curves were almost identical in both shape and trend.

The results of DSC were shown in Figure 3B. Two obvious heat absorption peaks for MT-GE and GE appeared. The first heat absorption peak was the denaturation temperature of MT-GE and GE, which were 80.21 °C and 66.99 °C, respectively. The Td of SPC/gelatin nanofibers prepared by Vahid et al. was 74.41 °C [40], which was lower than that of MT-GE (80.21 °C). The broad absorption peaks observed between 60 °C and 90 °C typically correspond to the endothermic dehydration process of bound water in gelatin. The results showed that the endothermic peak intensity of MT-GE in this range is higher than that of GE, indicating that the folic acid molecules (containing carboxyl and amino groups) form denser hydrogen bonds or electrostatic interactions with the gelatin molecular chains, thereby making the water binding more tightly and increasing the energy required for dehydration. The second heat absorption peak was the melting points of MT-GE and GE, which were 292.88 °C and 288.50 °C, respectively. At approximately 330 °C, a distinct endothermic peak was observed in the MT-GE sample. This is likely related to the dissociation of the folic acid-gelatin complex and the melting of the possible crystalline regions within the material. The peak shape was relatively sharp, indicating that the corresponding dissociation or melting process occurred relatively rapidly. It indicated that the introduction of a small amount of MTHF CAC (0.4%, *w*/*w*) effectively enhanced the thermal stability of the composite fibers through intermolecular interactions. Therefore, the thermal stability of MT-GE was higher than that of GE, which was probably due to the reaction between -OH in the MTHF CAC molecule and -COOH in the gelatin. The crosslinking between the molecules led to an increase in hydrogen bonds and van der Waals forces, which increased the enthalpy of the material [34,36,37,40,41,42,43,44]. The results were accordant to those achieved by TGA. The overall thermograms were similar because fish gelatin constituted the main matrix component, while the loading amount of MTHF CAC was only 0.4% (*w*/*w*). The differences in key thermal parameters confirmed the improved thermal stability of MT GE.

### 2.4. X-Ray Diffraction Analysis (XRD)

**Figure 4:**
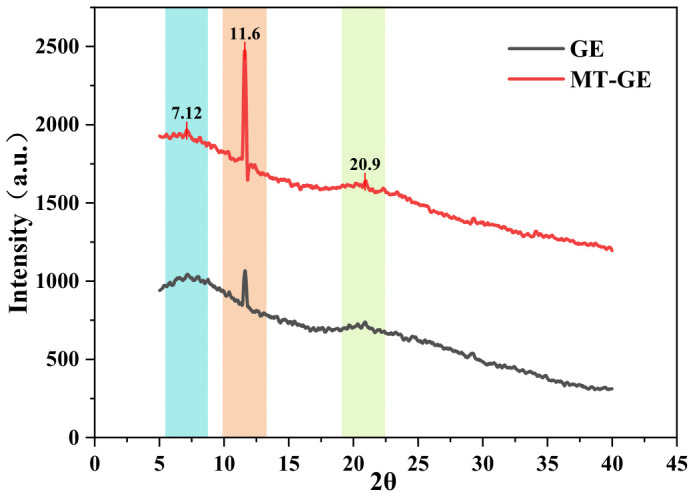
X-ray diffraction patterns of MT-GE and GE.

Figure 4 shows the X-ray diffraction patterns of MT-GE and GE. One distinct peak appeared at 2θ = 7.12°, which represented the helix conformation in gelatin [45]. The peak around 20.94° represented the amorphous region. With the conversion of collagen into gelatin, the original intact triple helix gradually disintegrated, and the amorphous structure gradually increased, resulting in a broad peak in the diffractogram [46]. The addition of MTHF CAC did not introduce new crystalline phases, as evidenced by the absence of additional peaks [47]. The gelatin nanofiber film showed a smaller peak at 2θ = 11.6°. However, the inclusion of MTHF CAC significantly amplifies the vibration of this peak, which might be due to the introduction of salt in the preparation solutions [48]. XRD is mainly used for the determination of crystals [49]. The diffraction peak at 11.6° indicated the change in crystal structure and the enhanced orderliness in the MT-GE composite material. The intensity of the peak at 11.6° was higher for MT-GE than for GE, indicating that the crystalline strength of the former was greater than that of the latter. The DSC measurement spectrum showed that the downward absorption peak at around 330 °C was related to the melting of the crystalline region. MT-GE had a higher downward absorption peak than GE. Therefore, the results of XRD were in accordance with those of the DSC.

### 2.5. Determination of the Loading Rate of MTHF CAC

**Figure 5:**
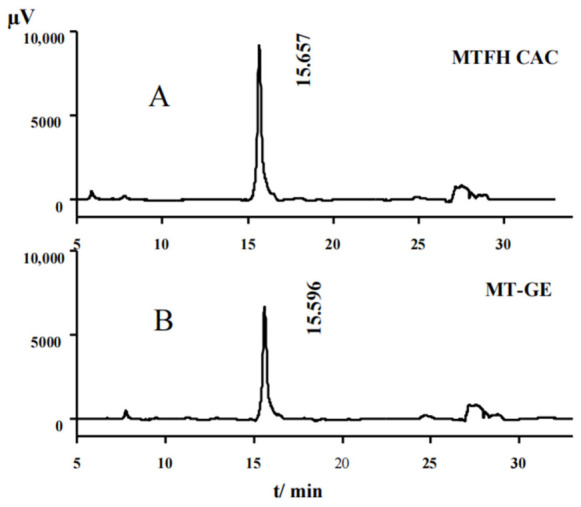
(**A**) An amount of 7.44 μg/mL of MTHF CAC solution. (**B**) MT-GE solution after deproteinization.

To investigate the stability of MTHF CAC in MT-GE, the loading rate of MTHF CAC was determined by HPLC. Figure 5 shows the HPLC profiles of MTHF CAC and MT-GE. MTHF CAC had strong UV absorption at 280 nm, and the standard curve could be obtained by measuring the peak area via HPLC as γ=21,759α+7386.3 (R2 = 0.9997, where γ is the peak area, and α is the concentration), with good linearity in the concentration range of 0.2–8 μg/mL. Its characteristic peak retention time was about 15.6 min (Figure 5A). MT-GE was measured by HPLC after pretreatment, and it also showed a characteristic peak at about 15.6 min (Figure 5B). The peak shape of MT-GE was good, and no other unknown peaks were observed near it. Therefore, the peak was determined as the characteristic peak of MTHF CAC. This finding demonstrated that MT-GE was successfully piggybacked on MTHF CAC. The calculated concentration of MTHF CAC was 6.37 μg/mL, indicating that the loading rate of MTHF CAC in MT-GE was 85.57%. MTHF CAC was not fully loaded in MT-GE, probably due to a small loss of MTHF CAC from the pre-treatment process before electrospinning. The choice of nanofiber matrix played a key role in the release effect [50]. This result showed that fish gelatin had an excellent carrier matrix piggybacking effect. The electrostatically spun MT-GE fiber membrane not only improved the thermal stability of MTHF CAC but also rapidly released the active ingredients of MTHF CAC from the carrier. It provided a good property basis for the application of MT-GE as a biomolecule material.

### 2.6. Cytocompatibility Evaluation

**Figure 6:**
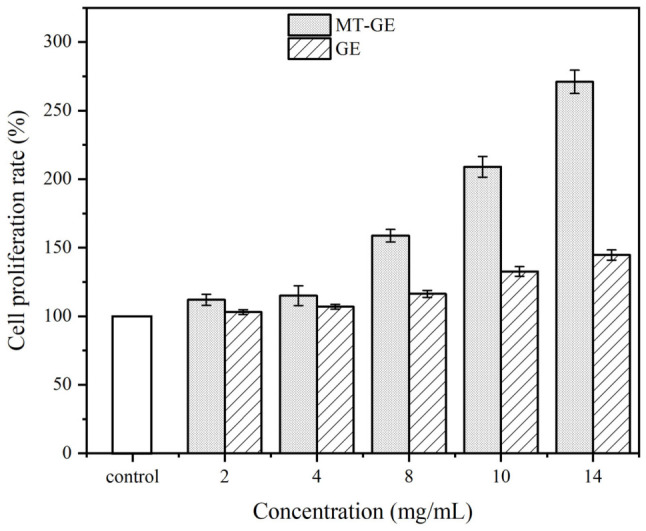
Effect of different concentrations of MT-GE and GE solution on cell proliferation.

Fibroblasts were commonly used in the evaluation and development of tissue engineering materials due to their rapid differentiation and regeneration properties [51,52]. Folate exhibited a significant cell proliferation-promoting effect, while fish gelatin, as a major component of the extracellular matrix (ECM), possessed excellent biocompatibility. The nanofiber membrane fabricated by combining the two components was expected to exert a synergistic promoting effect in the treatment of chronic non-healing wounds such as diabetic wounds. As a medical biomaterial, its cytotoxicity against fibroblasts was a core safety indicator that needed to be prioritized prior to clinical utilization. The in vitro cytotoxicity tests of MT-GE nanofibers toward NIH3T3 cells were evaluated using CCK-8 assays. Figure 6 showed that the cell viabilities for 24 h were over 100% at all test concentrations up to 14 mg/mL, revealing the non-toxicity of MT-GE toward cells. The cell viabilities at the concentration of 8 mg/mL MT-GE and GE solution was 158.8% and 116.3%, respectively. When the concentration reached 14 mg/mL, the cell proliferation rate of MT-GE was 1.87 times that of GE. It is well known that gelatin is a gold standard material for tissue engineering due to its good biological recognition. Due to the presence of arginine-glycine-aspartic acid sequences (RGD), this protein is able to improve cell adhesion, mimicking the major biochemical signals that are present in the cell microenvironment [53]. Folate played a vital role in the growth and repair of cells, aiding wound healing by synthesizing nucleic acids [22,24,54]. It could promote cell proliferation by upregulating the expression of proteins, such as Cyclin A2 and vascular endothelial growth factor (VEGF) [55]. Additionally, folic acid could also activate the Notch signaling pathway, which regulates cell proliferation, differentiation and apoptosis, leading to increased expression of receptors Notch1 and signaling molecules Hes5 and thereby promoting cell proliferation [56].

Fatma, etc., prepared a PVA/Gel nanofiber membrane and the proliferation rate of L929 cells was 82.99% [25]. The FCP scaffolds obtained by electrospinning 10% (*w*/*v*) collagen and 10% (*w*/*v*) PCL showed a 95% proliferation rate of HaCaT cells [27]. The proliferation rate of the SF/CL scaffolds prepared by e-spinning of collagen and filipin proteins was only 65% for NIH3T3 cells [26]. Therefore, the nanofiber membrane prepared from fish gelatin and MTHF CAC had better biocompatibility and could be an appropriate candidate for wound dressing.

## 3. Conclusions

In this study, MT-GE blends were prepared by electrospinning MTHF, CAC and gelatin in the aqueous system to obtain the nanofiber membrane. The FTIR spectrum showed the formation of hydrogen bonds between gelatin and MTHF CAC. The X-ray diffraction pattern indicated a slight enhancement in the crystallinity of MT-GE. The results of DSC and TGA showed that the thermal stability of MT-GE was higher than that of GE, which was in agreement with the results of XRD. MT-GE significantly enhanced NIH/3T3 cell proliferation, achieving a rate 2.71 times that of the GE control at 14 mg/mL.

Therefore, the MT-GE nanofiber membranes prepared from fish gelatin and MTHF CAC had great potential as a candidate for wound dressing. Future work is being carried out on the impact of this material on cell differentiation and attachment, its effect on wound healing, and the release effect of MTHF CAC.

## 4. Materials and Methods

### 4.1. Materials

Fish gelatin was provided by the Collagen Laboratory of Jiangsu Ocean University, Lianyungang, China. 6S-5-methyltetrahydrofolate calcium salt crystal form C (MTHF CAC) was provided by Lianyungang Jinkang Hexin Pharmaceutical Co., Ltd., Lianyungang, China. NIH-3T3 cells were purchased from the Kunming Cell Bank of the Typical Culture Collection Committee of the Chinese Academy of Sciences, Kunming, China. Trypsin-EDTA (0.25%) containing phenol red, purchased from GIBCO, Shanghai, China. CCK-8 kit, penicillin/streptomycin solution, DMEM medium, fetal bovine serum, phosphate-buffered solution, and other reagents were purchased from Sangon Biotech Co., Ltd., Shanghai, China.

### 4.2. Methods

#### 4.2.1. Preparation of Electrospinning Solution and Electrospinning

Nanofibers were prepared using an electrospinning device (DP30, Yunfan Technology Co., Ltd., Tianjin, China). A gelatin solution was prepared according to the method of Songchotikunpan et al. [17]. The gelatin was dissolved in a 40% (*v*/*v*) acetic acid aqueous solution to obtain the gelatin solutions at a concentration of 15% (*w*/*v*). 4 mg MTHF CAC was mixed into the gelatin solutions with the final concentration of 0.4%(*w*/*w*). The solution was loaded into a 5 mL plastic syringe with a stainless steel needle and pumped at a flow rate of 0.315 mL/h. The metal needle size of the syringe was 18 G with a nominal inner diameter of 0.84 mm and an outer diameter of 1.27 mm. A positive voltage of 20 KV was used, and the tip-to-collector distance was 15 cm. The collection time was fixed at 127 min. All measurements were carried out in triplicate at room temperature (25 ± 1 °C). Gelatin nanofibers (GE) were prepared by the same method as a control. Supplementary Figure S2 showed the electrospinning process of the gelatin solution containing MTHF CAC (MT-GE).

#### 4.2.2. Rheological Properties of Electrospun Solutions

The rheological properties of the spinning solution were determined using a rheometer (MCR102, Anton Paar, Ostfildern, Germany) at 25 ± 1 °C with a rotor diameter of 25 mm. Each sample was equilibrated for 5 min before testing. To avoid evaporation of the sample, light silicone oil was applied to the gap between the rotor and the measuring disk [57]. The shear rate was set in the range of 1–1000 s^−1^ [58].

#### 4.2.3. Scanning Electron Microscopy (SEM)

Both samples of gelatin electrospun film (GE) and MT-GE were surface sprayed with gold by an ion sputtering instrument (SBC12, Beijing CSCI Technology Co., Ltd., Beijing, China). The samples were morphologically characterized by cold field emission scanning electron microscopy (Regulus8100, Hitachi Limited, Singapore). Origin 2018 software was used to statistically analyze the diameter distribution of nanofibers.

#### 4.2.4. Fourier Transform Infrared Spectroscopy (FTIR)

The electrospun films were scanned and analyzed by Fourier infrared transform spectroscopy (FT-IR Spectrometer, Perkin Elmer, Boston, MA, USA). The scanning range was 4000 to 400 cm^−1^ with an interval of 1 cm^−1^ and a resolution of 4 cm^−1^ [59,60].

#### 4.2.5. Differential Scanning Calorimetry and Thermogravimetry Analysis (DSC and TGA)

The thermal properties of the nanofiber membranes were analyzed with a simultaneous thermal analyzer (STA8000, Perkin Elmer, USA) to determine the DSC and TGA parameters of different samples. The samples were weighed in a crucible and warmed up from 30 °C to 400 °C at a ramp rate of 10 °C/min in a gaseous environment with a nitrogen flow rate of 20 mL/min [61].

#### 4.2.6. X-Ray Diffraction Analysis (XRD)

An X-ray diffractometer (X’Pert Powder, PANalytical B.V., Almelo, The Netherlands) was used to analyze the crystallinity variation in MT-GE. The XRD system was operated at a scan range of 2θ = 5–40°, 20 KV, and 5 mA, Kα (λ = 1.5418), a rate of 4°/min, and a step of 0.02° [61,62].

#### 4.2.7. Determination of the Loading Rate of MTHF CAC

The MT-GE was dissolved in 1 mL of ultrapure water. Vc was added at the ratio of 1:1 (*w*/*w*) of MTHF-CAC and dissolved thoroughly. The solution was added to the extractant (1-Butanol:Trichloromethane = 1:4) at a ratio of 9:1. The protein was removed by centrifugation at 6000 rpm/min for 10 min, and the supernatant was then filtered through a 0.22 μm membrane [63]. MTHF CAC retention rate was determined by HPLC. The chromatographic column of HPLC was a C18 column, 250 mm × 4.6 mm, with a particle size of 5 μm. The mobile phase was a 35% methanol solution at a flow rate of 1.1 mL/min. The detection wavelength was 280 nm. The injection volume was 10 μL. The column temperature was 32 °C. And the acquisition time was 33 min.

#### 4.2.8. Cytocompatibility Evaluation

Samples were tested for cytotoxicity. NIH3T3 cells were cultured in complete medium (89% (*v*/*v*) DMEM, 10% (*v*/*v*) fetal bovine serum (FBS), and 1% (*v*/*v*) penicillin/streptomycin) at 37 °C in 5% CO_2_. NIH-3T3 cells growing at 80–90% confluence at the bottom of T25 culture flasks were digested with trypsin. A cell suspension of approximately 5 × 104 cells/mL was prepared. A 96-well plate was taken and 100 μL of cell suspension was added to the sample and control groups, with 3 replicate wells for each group. After 24 h of incubation, 100 μL of the sample to be tested (sample group) or 100 μL of complete medium (control group) was added to each well. Samples were prepared in complete medium with MT-GE solutions at concentrations of 2, 4, 8, 10, and 14 mg/mL, respectively. After 24 h of incubation, 10 μL CCK-8 was added. After 1 h of incubation, the OD value was measured at 450 nm (complete medium was used as the blank group). The cell proliferation rate was calculated using the standard CCK-8 formula which is indicated in the Supplementary Materials.

#### 4.2.9. Statistical Analysis

All data graphs were designed by Origin 2018 (Microcal, USA) and the data were statistically analyzed by IBM SPSS Statistics 20 (IBM, USA).

## Data Availability

The raw data supporting the conclusions of this article will be made available by the authors on request.

## Supplementary Materials

The following supporting information can be downloaded at: https://www.mdpi.com/article/10.3390/gels12070563/s1, Figure S1: FTIR scan spectra of MT-GE and GE; Figure S2: The electrospinning process of MT-GE.

## Abbreviations

The following abbreviations are used in this manuscript:

MT-GE	Nanofiber Membrane Composed of MTHF CAC and Fish Gelatin
MTHF CAC	6S-5-Methyltetrahydrofolate Calcium Salt Crystal Form C
GE	Gelatin Nanofiber (Control Group)
SEM	Scanning Electron Microscopy
FTIR	Fourier Transform Infrared Spectroscopy
DSC	Differential Scanning Calorimetry
TGA	Thermogravimetric Analysis
XRD	X-Ray Diffraction
HPLC	High-Performance Liquid Chromatography
CCK-8	Cell Counting Kit-8
DMEM	Dulbecco’s Modified Eagle Medium
FBS	Fetal Bovine Serum
PBS	Phosphate-Buffered Solution
NIH/3T3	Mouse Embryonic Fibroblast Cell Line
NO	Nitric Oxide
RGD	Arginine-Glycine-Aspartic Acid Sequence
VEGF	Vascular Endothelial Growth Factor
UV	Ultraviolet
