# Supplementary material for: Study on the Preparation and Properties of MT-GE (6S-5-Methyltetrahydrofolate Calcium Salt Crystal Form C-Gelatin) Nanofiber Membrane
Source: Gels. 2026 Jun 25;12(7):563. doi: 10.3390/gels12070563 (PMC13408474; doi:10.3390/gels12070563)
Supplement: Supplementary file 1 [file gels-12-00563-s001.zip › gels-4266842-supplementary.pdf]

Supplementary Information (SI)

Study on the Preparation and Properties of MT-GE(6S-5-Methyltetrahydrofolate Calcium Salt Crystal Form C-Gelatin) Nanofiber Membrane

Yuhang Wang [1], Ke Wang [1,2], Mochi Zhu [1], Yu Liu [1,2], Tianyue Xu [1], Rui Duan [3] and Junjie Zhang [1,2*]

1 School of Ocean Food and Bioengineering, Jiangsu Ocean University, Lianyungang 222005, China

2 Jiangsu Institute of Marine Resources Development, Jiangsu Ocean University, Lianyungang 222005, China

3 School of Marine Science and Fisheries, Jiangsu Ocean University, Lianyungang 222005, China

* Correspondence: zhangjj916@jou.edu.cn

This file includes:

A. Supplementary figure (Figure S1、Figure S2)

B. Supplementary equation (Equation S1)

A. Supplementary figure

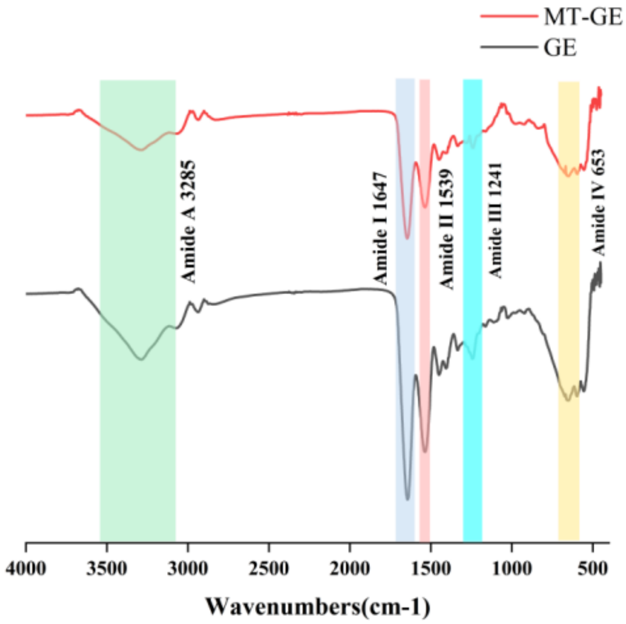

Figure S1. FTIR scan spectra of MT-GE and GE.

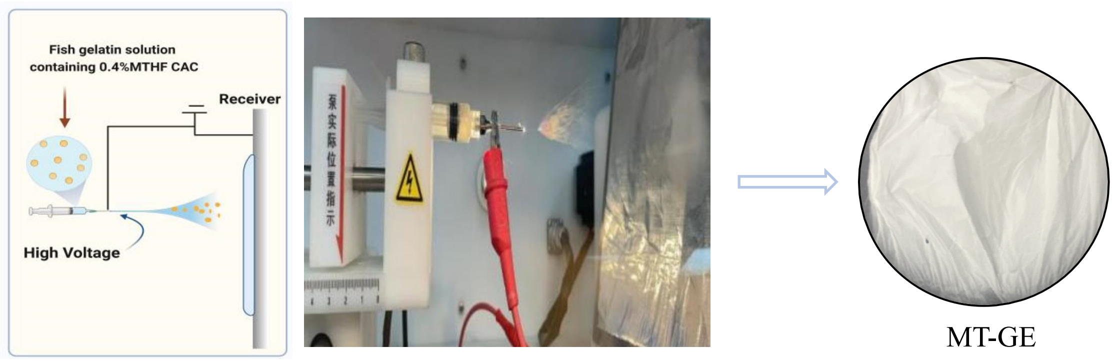

Figure S2.The electrospinning process of MT-GE.

B. Supplementary equation

Supplementary Materials: Cytocompatibility Evaluation (CCK-8 Assay)

Equation S1. Calculation of cell proliferation rate.

The percentage of cell proliferation was determined using the following standard formula for CCK-8 assays:

$$Cell\ proliferation\ rate(\%) = \frac{(A - A_0)}{(A_1 - A_0)} \times 100\%$$

The formula:

A - Absorbance at 450 nm for the experimental group;

A0 - Background absorbance (blank control);

A1 - Absorbance reading for the untreated control group.
